# To Prevent Sleeping in Church

**Published:** 1856-07

**Authors:** 


### TO PREVENT SLEEPING IN CHURCH.

Too many of our clergy have settled down into a conviction, that some how or other, their sermons in the aggregate, in the long run, will have a converting effect on the minds of their habitual hearers; and not being worked up into any expectation of an immediate conversion, their pulpit efforts, in the main, have degenerated into a tread-mill monotony; and it cannot but be expected that under such discourses, an active business man, who has been stirred up all the week by the merry jingle of dollars, can do otherwise than grow drowsy. He may be ashamed of it at the time; he may have conscience enough to lament it, and a will too, to fight against it, but the power, where is it? And any vain effort he may make to wake up, and get at the "*thread of the discourse,*" only diverts his attention. For our part, we know of no available method of keeping wide-awake, within the sound of a dull sermon than this,

TAKE A NAP BEFORE YOU GO.

